# Da Cheng Qi Decoction Alleviates Cerulein-Stimulated AR42J Pancreatic Acinar Cell Injury via the JAK2/STAT3 Signaling Pathway

**DOI:** 10.1155/2021/6657036

**Published:** 2021-04-09

**Authors:** Zehua Zhou, Ying Chen, Wenmin Dong, Rui An, Kun Liang, Xinhong Wang

**Affiliations:** ^1^Shanghai University of Traditional Chinese Medicine, Shanghai 201203, China; ^2^Shanghai Traditional Chinese Medicine Integrated Hospital, Shanghai University of Traditional Chinese Medicine, Shanghai 200082, China

## Abstract

**Background:**

Acute pancreatitis (AP) is a common acute abdomen inflammation, characterized by the dysregulation of digestive enzyme production and secretion. Many studies have shown that Da Cheng Qi Decoction (DCQD) is a secure, effective prescription on AP. In this study, cerulein-stimulated AR42J cells damage model was established to further explore the feasibility and underlying mechanism of DCQD as a potential inhibitor of JAK2/STAT3 pathway for the treatment of AP.

**Methods:**

Cell viability of DCQD was measured using a cell counting Kit-8 assay. Pancreatic biochemical markers such as amylase, lipase, and C-reactive protein production were measured by assay kits, respectively. Cytokines (TNF-*α*, IL-6, IL-10, and IL-1*β*) were assayed by ELISA. Protein location and protein expression were detected by immunofluorescence staining and Western blotting, respectively. Gene expression was assessed by real-time PCR. For mechanistic analysis of the effect of DCQD on JAK2/STAT3 signaling pathway, selective JAK2 inhibitor (Fedratinib) and STAT3 inhibitor (Stattic) as well as STAT3 activator (Garcinone D) were used.

**Results:**

DCQD protected cells by regulating cerulein-induced inflammation and reducing the secretion of pancreatic biochemical markers. Moreover, DCQD could not only inhibit the nuclear translocation of p-STAT3, but also decrease the mRNA expression of JAK2 and STAT3 as well as the ratio of p-JAK2/JAK2 and p-STAT3/STAT3 in protein level. Additionally, DCQD could regulate the mRNA and protein expression of JAK2/STAT3 downstream effectors, Bax and Bcl-XL. The activated effect of cerulein on JAK2/STAT3 pathway was also reversed by JAK2 inhibitor Fedratinib or STAT3 inhibitor Stattic. And the overexpression of JAK2/STAT3 pathway, via STAT3 activator Garcinone D, did exert damage on cells, which bore a resemblance to cerulein.

**Conclusion:**

The activation of JAK2/STAT3 pathway may play a key role in the pathogenesis of cerulein-stimulated AR42J pancreatic acinar cell injury. DCQD could improve inflammatory cytokines and cell injury, which might be mediated by suppressing the activation of JAK2/STAT3 signaling pathway.

## 1. Background

Acute pancreatitis (AP) is a common acute abdomen inflammation, of which severe complication involves systemic inflammatory response syndrome (SIRS) and multiple organ dysfunction syndromes (MODS) [1, 2]. The incidence and prevalence of AP have been increasing worldwide and have a great influence on life quality and work ability. Growing evidences have shown that several factors, including cholelithiasis, alcoholism, and smoking, increase the incidence and mortality burden [3]. Currently, due to the lack of available medicines, the mainstay of treatment for AP is based on surgery [4]. However, poor surgical prognosis and high recurrence rate have made the operation result unsatisfactory.

Nowadays, Traditional Chinese Medicine (TCM) has become one of the most popular complementary and alternative therapies in the world. Da Cheng Qi Decoction (DCQD), a famous formula recorded in Shang Han Lun, consists of *Rheum officinale* Baill. (Da Huang), *Magnolia officinalis* Rehder & E.H.Wilson (Hou Pu), *Fructus Aurantii Immaturus* (Zhi Shi), and *Natrii Sulfas* (Mang Xiao). Over the years, many studies have shown that DCQD is a secure, effective drug on AP [5, 6].

Presently, more and more clinical studies have proven that the levels of a few pro- and anti-inflammatory cytokines, such as tumor necrosis factor-*α* (TNF-*α*), interleukin (IL)-6, and interleukin (IL)-10, are elevated early in patients with AP [7]. Determining the severity of inflammatory reaction at admission does contribute to predicting clinical outcome in AP. On the other hand, as one of the principal signaling pathways for cytokines and growth factors, Janus kinase 2 signal transducers and transcription 3 (JAK2/STAT3) signaling pathway is essential for the innate immunity and suppression of inflammation [8]. In addition, many researchers suggested that JAK2/STAT3 signaling pathway acts as an important role in the pathogenesis and development of AP [9, 10]. Inflammatory cytokines can participate in the pathogenesis of AP by activating JAK2/STAT3 pathway [11]. At the same time, many drugs can treat AP through JAK2/STAT3 pathway [12, 13]. Moreover, based on the enrichment results of KEGG (Kyoto Encyclopedia of Genes and Genomes) in network pharmacology and proteome research in our previous research, JAK2/STAT3 signal pathway may be the key of DCQD in interfering with AP.

Although a previous study sporadically showed that DCQD could regulate inflammatory cytokines and intestine injury in rats with severe acute pancreatitis via JAK2/STAT3 signaling pathway [14], the potential mechanism of DCQD participating in AP through JAK2/STAT3 is not clear. A simple and feasible in vitro model of AP should be considered. In this study, cerulein-induced AR42J cells damage model was established to further explore the feasibility and underlying mechanism of DCQD as a potential JAK2/STAT3 inhibitor for the treatment of AP (additional file, Figure 1).

## 2. Methods

### 2.1. Preparation of DCQD

Da Huang, Hou Pu, Zhi Shi, and Mang Xiao were purchased from Shanghai Kangqiao Chinese Medicine Pieces Co., Ltd. (Shanghai, China). The raw materials were qualified according to analysis listed in the Pharmacopoeia of the People's Republic of China 2015 Edition and authenticated by Pharmacist Yanjun Cheng (Shanghai Hongqiao Chinese Medicine Pieces Co., Ltd., Shanghai, China). Da Huang, Hou Pu, Zhi Shi, and Mang Xiao were weighed according to the ratio of 12 : 24 : 12 : 9.Briefly, Hou Pu and Zhi Shi were boiled twice. First, Hou Pu and Zhi Shi were dipped in cold water at a ratio of 1 : 6 (W/V) for 30 min and then boiled for 30 min. Da Huang was soaked in the hot filtrate for 10 min and then filtered again. Next, the residues of the first decoction were further boiled with 4 times water for 20 min. Da Huang was mixed as mentioned previously. Mang Xiao was dissolved in the mixture of these two filtrates while hot. Condensed to an appropriate volume, 95% ethanol was added two times to 60% and 75% ethanol concentration, respectively. The solution was filtered after storing in a refrigerator at 4°C for 24 h. Finally, the aqueous extract was concentrated by reduced-pressure evaporation to 150 mL (equal to 1 g/mL of dried herbs).The yield of DCQD was up to 15.3%. The fingerprint chromatogram of DCQD has been identified by using a HPLC method (additional file, Figure 2), in which the major peaks were identified by comparing both the retention times of both DCQD and the reference standards. Notably, 9 compounds in DCQD, viz., [1] naringin, [2] hesperidin, [3] aloe-emodin, [4] rhein B, [5] honokiol, [6] magnolol, [7] emodin, [8] chrysophanol, and [9] physcion, were properly identified. The results of fingerprint chromatogram of several batches of DCQD showed good repeatability. The extract of DCQD was diluted 20 times by culture media as the stock solution for the following experiment (the concentration was recorded as C).

### 2.2. Cell Culture

AR42J rat pancreatic acinar cells were purchased from the American Type Culture Collection (Manassas, VA, USA). Cells were cultured in RPMI-1640 (Hyclone Labs, UT, USA) medium containing 10% fetal bovine serum (FBS) (GIBCO, NY, USA) and 1% penicillin-streptomycin solution (100 X) (Solarbio Science & Technology Co., Ltd., Beijing, China) at 37°C in a humidified 5% CO_2_ atmosphere.

### 2.3. Cell Viability Assay

For detection of DCQD cytotoxicity and cell viability, cell counting kit‐8 (CCK‐8) assay was applied in this study. Procedures were as follows. Firstly, AR42J cells were incubated in each well of a 96-well plate at a density of 4 × 10^3^ cells/well and grown for 12 hours. Thereafter, cells were pretreated with different concentrations of DCQD (C, C/2, C/3, C/4, C/5, C/10) for 2 h. Secondly, the CCK‐8 solution (Signalway Antibody LLC, Maryland, USA) was added to each well. After incubation for 1 h, absorbance for detecting cell viability was read at 450 nm on a microplate reader (iMark680; Bio-Rad Laboratories, Inc.) and converted to cell numbers with the standard curve. To observe the morphological change, the cells were observed under an optical microscope (XDS-500C, Peikon, Shanghai).

### 2.4. Cell Treatment

Based on the CCK-8 results, different doses of DCQD (C/6, C/5, and C/4) were selected for the further study.

To explore the amelioration of DCQD on AR42J cells injury induced by cerulein (CER), cells were divided into the following groups: control: cells were cultured in medium and treated with phosphate buffered saline (PBS); model, cells were stimulated with CER (10^−7^M) (Shanghai Yuanye Biotechnology Co., Ltd., Shanghai, China) as previous prescribed [15]; low dose group (LG), medium dose group (MG), and high dose group (HG): cells were treated with DCQD (C/6, C/5, and C/4, respectively) for 2 h before the stimulation with CER (10^−7^M).

To further verify the effect of DQCD on JAK2/STAT3 signaling pathway, cells were divided into the following groups: control: cells were cultured in medium and treated with PBS; model: cells were stimulated with CER (10^−7^M); DCQD group (DG): cells were treated with DCQD (C/4) without stimulation with CER (10^−7^M); treatment group (TG): cells were treated with DCQD (C/4) for 2 h before the stimulation with CER (10^−7^M).

To study whether DCQD ameliorated CER-induced AR42J cells injury by suppressing the activation of JAK2/STAT3 signaling pathway, cells were divided into the following groups: control: cells were cultured in medium and treated with PBS; model: cells were stimulated with CER (10^−7^M); DCQD group (DG): cells were treated with DCQD (C/4) without stimulation with CER (10^−7^M); treatment group (TG): cells were treated with DCQD (C/4) for 2 h, prior to CER (10^−7^M) stimulation; Fedratinib group (FG): cells were treated with Fedratinib (3 nM) (JAK2 inhibitor) for 2 h, prior to CER (10^−7^M) stimulation; Stattic group (SG): cells were treated with Stattic (5 *μ*M) (STAT3 inhibitor) for 2 h, prior to CER (10^−7^M) stimulation; Garcinone D group (GG): cells were stimulated with Garcinone D (10 *μ*M) (STAT3 activator); DCQD + Garcinone D group (D + GG): cells were treated with DCQD (C/4) for 2 h, prior to Garcinone D (10 *μ*M).

The cells were harvested 8 h after the onset of treatment and stored at −20°C for later experiments. Concentrations of Fedratinib (3 nM), Stattic (5 *μ*M), and Garcinone D (10 *μ*M) were adapted from previous studies showing inhibition or activation of JAK2/STAT3 signaling pathway in vitro [16–18].

### 2.5. Measurement of Amylase, Lipase, and C-Reactive Protein Production

Amylase assay kit (Nanjing Jiancheng Bioengineering Institute, Nanjing, China), lipase assay kit (Nanjing Jiancheng Bioengineering Institute, Nanjing, China), and C-reactive protein assay kit (Nanjing Jiancheng Bioengineering Institute, Nanjing, China) were used to determine the activity of amylase (660 nm), lipase (420 nm), and C-reactive protein (570 nm), respectively, according to the instructions provided by the manufacturer.

### 2.6. Enzyme-Linked Immunosorbent Assay (ELISA)

Inflammatory cytokines TNF-*α*, IL-1*β*, IL-6, and IL-10 production in the supernatant of treated cells was measured using ELISA kits (Nanjing Jiancheng Bioengineering Institute, Nanjing, China) by following the manufactures' protocols. In the end, the absorbance data of each well was measured at 450 nm. TNF-*α*, IL-1*β*, IL-6, and IL-10 concentration (ng/L) was detected using standard purified recombinant cytokine.

### 2.7. Immunofluorescence Staining

Cells of each group were washed three times with PBS and fixed with formaldehyde for 30 min at room temperature. Then, cells were permeabilized with 0.5% Triton X-100 (Solarbio Science & Technology Co., Ltd., Beijing, China) in PBS for 10 min. After 1 h in 1% BSA (Solarbio Science & Technology Co., Ltd., Beijing, China), the cells were incubated at 4°C for 12 hours with rabbit anti-p-STAT3 (1 : 1000). Thereafter, cells were briefly washed with PBS and incubated with an Alexa Fluor 488 conjugated goat anti-rabbit IgG (H + L) (Beyotime, Shanghai, China) antibody at room temperature for 1 h. The nuclei of cells were stained with Hoechst 33342 for 10 min. Fluorescence microscope was used to capture the images. The rabbit anti-p-STAT3 (catalog number: ab76315) was obtained from Abcam (Abcam, Cambridge, UK).

### 2.8. Real-Time PCR Analysis

Total RNA was isolated from treated cells by using Trizol Reagent (Invitrogen, CA, USA) and was treated with RNase-free DNase. Reverse transcription was performed with the cDNA reverse transcription kit (Fermentas, MA, USA). For real-time PCR reactions, amplification mixture (25 *μ*L) contained 1 *μ*L of primer mix, 2 *μ*L of cDNA template, 12.5 *μ*L of SYBR green Mix (Thermo Scientific Molecular Biology, MA, USA), and 9.5 *μ*L of ddH_2_O. Specific mRNA quantification was performed on ABI 7300 Real-Time PCR instrument (Applied Biosystems, CA, USA), according to the manufacturer's guidelines. The data were analyzed by use of ABI Prism 7300 Sequence Detection System software (Applied Biosystems, CA, USA). Expression levels of the mRNAs of interest were normalized to those of endogenous reference gene glyceraldehyde-3-phosphate dehydrogenase (GAPDH). The expression of mRNAs relative to GAPDH was calculated by the 2^−∆∆Ct^ method. All steps were performed under RNase-free conditions. Primers for JAK2, STAT3, Bcl-XL, Bax, and GAPDH in the reaction are listed in Table 1.

### 2.9. Western Blot Analysis

Treated cells of each group were lyzed in RIPA buffer (Shanghai JRDUN Biotechnology Co., Ltd., Shanghai, China) containing both protease and phosphatase inhibitor mixture to extract whole cell protein. We used BCA assay kit (Thermo Scientific Molecular Biology, MA, USA) to quantify the protein levels. The proteins were separated by SDS-PAGE and transferred to polyvinylidene fluoride (PVDF) membranes. The nonspecific sites on each blot were blocked with 5% skim milk for 12 hours at 4°C. Target proteins were detected by corresponding primary antibodies. After overnight incubation with the primary antibody, the membranes were washed 3 times and incubated with anti-rabbit IgG-HRP conjugated secondary antibody. The protein bands were imaged using ECL and normalized against GAPDH by Tanon-5200. Antibodies were used at the following concentrations: anti-JAK2/p-JAK2 1 : 5000, anti-STAT3 1 : 1000, anti-p-STAT3 1 : 20000, anti-Bax 1 : 1000, anti-Bcl-XL 1 : 1000, GAPDH 1 : 2000, and anti-rabbit IgG-HRP conjugated secondary antibody 1 : 1000. Sources of antibodies were as follows: Rabbit monoclonal [EPR108(2)] antibodies against JAK2 (catalog number: ab108596), Rabbit monoclonal [E132] antibodies against JAK2 (phospho Y1007 + Y1008) (catalog number: ab32101), Mouse monoclonal [9D8] antibodies against STAT3 (catalog number: ab119352), Rabbit monoclonal [EP2147Y] antibodies against STAT3 (phospho Y705) (catalog number: ab76315), Rabbit monoclonal [E18] antibodies against Bcl-XL (catalog number: ab32370), and Rabbit monoclonal [E63] antibodies against Bax (catalog number: ab32503), which were from Abcam (Abcam, Cambridge, UK); GAPDH (catalog number: 5174) was from CST (Cell Signaling Technology, Inc., MA, USA); anti-rabbit IgG-HRP conjugated secondary antibody (catalog number: A0208) was from Beyotime (Beyotime, Shanghai, China).

### 2.10. Statistical Analysis

Each experiment above was repeated in triplicate to ensure quantitative accuracy. Statistical analysis was performed by SPSS 22.0 (SPSS Inc., Chicago, IL, USA). All experimental data were presented as the mean ± standard deviation (SD) and analyzed for statistical significance by one-way ANOVA. Values of *P* < 0.05 and *P* < 0.01 were defined as significant and very significant difference.

## 3. Results

### 3.1. Cytotoxicity and Cell Proliferation in AR42J Cell Exposed to DCQD

**Figure 1:**
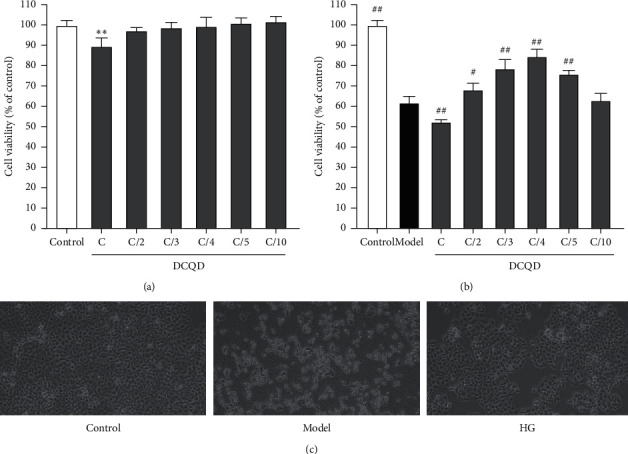
Cytotoxicity and cell proliferation in AR42J cells exposed to DCQD. (a) AR42J cells were incubated with DCQD at the concentrations of C, C/2, C/3, C/4, C/5, and C/10 for 2 h. Cell viability was measured by the CCK-8 assay. (b) AR42J cells were pretreated with DCQD at the concentrations of C, C/2, C/3, C/4, C/5, and C/10 for 2 h, and then cells were stimulated with CER (10^−7^M) for 8 h. Cell viability was measured by the CCK-8 assay. (c) The morphological changes of cells were assessed by using an optical microscope (400×). Results are expressed as the mean ± SD, *n* = 3. Micrographs from one representative experiment out of three independent experiments are shown. All bar graphics:^*∗*^*P* < 0.01 vs. the control group; ^##^*P* < 0.01 vs. the model group.

DCQD significantly reduced cell viability at doses of C (*P* < 0.01) (Figure 1(a)) and showed no evident cytotoxic effects from concentration C/2. Therefore, a further study about the effect of DCQD on reversing CER-induced cell proliferation inhibition was carried out. Cells were preincubated with different concentrations of DCQD (C, C/2, C/3, C/4, C/5, and C/10) for 2 h and then stimulated with CER. As shown in Figure 1(b), CER stimulation significantly reduced cell viability (*P* < 0.01), and DCQD preincubation increased cell viability in an approximately dose-dependent manner. Cell viability was highest at concentration of C/4, followed by C/5. We speculated that the most effective concentration of DCQD in cells was C/4. As a result, we selected this concentration as well as C/5 and C/6 for subsequent experiments to observe the concentration-dependent manner. In addition, under the optical microscope (Figure 1(c)), the AR42J cells in the control group had normal morphology and structure, gathered in clumps or lumps, and could produce appropriate amount of secretions. However, compared to control group, the AR42J cells in the model group showed severe inflammatory reaction, of which density decreased and volume increased. More suspended cell fragments and necrotic cells could be founded. After the pretreatment of DQCD (C/4), the morphology and structure of cells tended to be normal. In a word, DCQD could alleviate cell injury induced by CER.

### 3.2. Effects of DQCD on the Reflective Markers of Severity of AP

**Figure 2:**
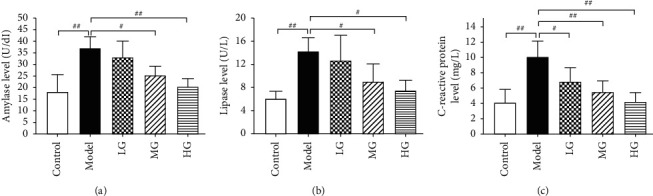
Effects of DQCD on the reflective markers of AP. The releases of amylase, lipase, and C-reactive protein were detected by amylase assay kit (a), lipase assay kit (b), and C-reactive protein assay kit (c), respectively. Data are presented as the means ± SD, *n* = 3. All bar graphics: ^#^*P* < 0.05, ^##^*P* < 0.01 vs. the model group.

In previous studies, the release of amylase has been used as a marker for monitoring models of AP, while lipase and C-reactive protein are potentially useful as markers of clinical severity of AP [19–21]. The results showed that CER induction markedly increased the levels of amylase (Figure 2(a)), lipase (Figure 2(b)), and C-reactive protein (Figure 2(c)) compared to control group (*P* < 0.01). In contrast, the levels of amylase, lipase, and C-reactive protein were significantly decreased in DCQD pretreatment groups (*P* < 0.05 or *P* < 0.01), demonstrating a concentration-dependent response.

### 3.3. Effects of DQCD on the Production of Inflammatory Cytokines

**Figure 3:**
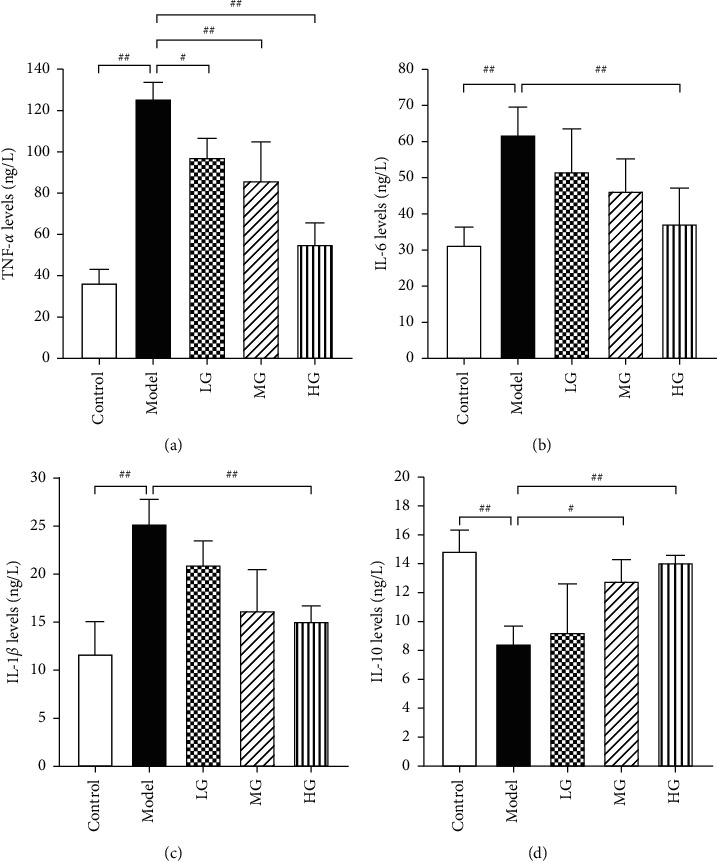
Effects of DQCD on the production of inflammatory cytokines. Inflammatory factors TNF-*α* (a), IL-6 (b), IL-10 (c), and IL-1*β* (d) in cellular supernatants were assayed by ELISA. Data are presented as the means ± SD, *n* = 3. All bar graphics: #*P* < 0.05, ##*P* < 0.01 vs. the model group.

Changes in cytokine production were consistent with the inflammatory state of AP. ELISA was utilized to determine the production of inflammatory cytokines such as TNF-*α*, IL-1*β*, IL-10, and IL-6 in AR42J cells. It was shown that CER triggered a significant elevating secretion of TNF-*α* (Figure 3(a)), IL-6 (Figure 3(b)), and IL-1*β* (Figure 3(c)) in AR42J cells compared with that in control group (*P* < 0.01). DCQD significantly decreased the level of IL-6, IL-1*β*, and TNF-*α* in a concentration-dependent manner (*P* < 0.05 or *P* < 0.01). On the other hand, a significant decrease in production of IL-10, a kind of anti-inflammatory cytokine, was noted in cells stimulated with CER compared to control group (*P* < 0.01) (Figure 3(c)). DCQD significantly increased the level of IL-10 in MG and HG group (*P* < 0.05 and *P* < 0.01, respectively). Overall, these results suggested that DCQD successfully protected against CER-induced AR42J cell injury and inflammation.

### 3.4. DCQD Suppressed JAK2/STAT3 Signaling Pathway in a Concentration-Dependent Manner

**Figure 4:**
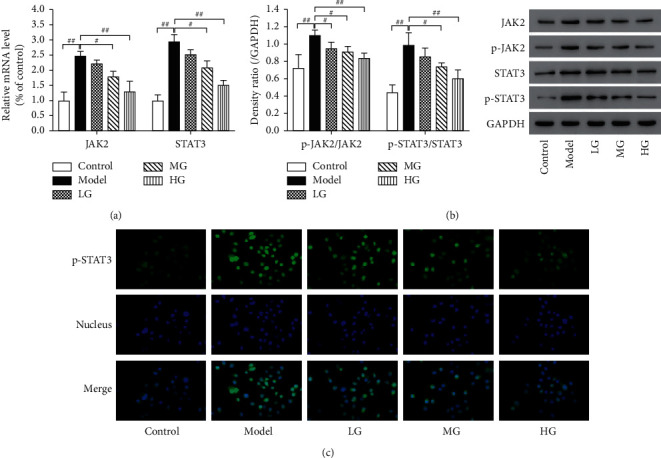
DCQD suppressed JAK2/STAT3 signaling pathway in a concentration-dependent manner. (a) Real-time PCR was performed to detect the mRNA expression of JAK2 and STAT3. (b) Western blot was performed to detect the protein level of JAK2, p-JAK2, STAT3, and p-STAT3. (c) Immunofluorescence staining (400×) was performed to detect the location of p-STAT3 in cells. GAPDH was as the loading control for band density normalization. Data are presented as the means ± SD, *n* = 3.Immunofluorescence micrographs from one representative experiment out of three independent experiments are shown. All bar graphics: #*P* < 0.05, ##*P* < 0.01 vs. the model group.

The effect of DCQD on JAK2/STAT3 signaling pathway in AR42J cells stimulated with CER was investigated. As illustrated in Figure 4(a), the mRNA expression of JAK2 and STAT3 was significantly increased in acinar cells treated with CER compared to control group (*P* < 0.01). Pretreatment of cells with DCQD decreased the mRNA expression of JAK2 and STAT3 in a dose-dependent manner (*P* < 0.05 or *P* < 0.01). Western blot analysis (Figure 4(b)) showed that the protein expression of p-JAK2 and p-STAT3 in model group was significantly increased compared to control group (*P* < 0.01), while their expression was significantly downregulated by DCQD, also in a dose-dependent manner (*P* < 0.05 or *P* < 0.01). Immunofluorescence analysis was performed for the location of p-STAT3 in cells. Generally speaking, p-STAT3 staining was mainly present in the cytoplasm. Once JAK2/STAT3 was activated, p-STAT3 would translocate to the nucleus [22]. Results confirmed that p-STAT3 protein was highly expressed in the nucleus of cells in model group, while its expression was suppressed in the nucleus of cells treated with DCQD (Figure 4(c)). All results showed that DCQD may be beneficial in AP by inhibiting the activation of JAK2/STAT3 signaling in pancreatic acinar cells.

### 3.5. Effect of DCQD on Downstream Effectors in the JAK2/STAT3 Pathway

**Figure 5:**
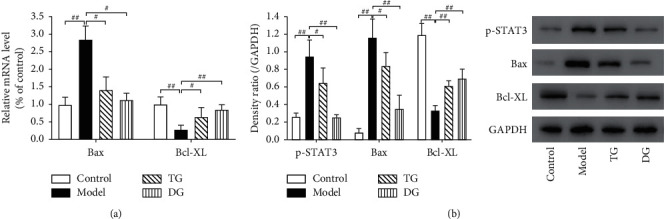
Effect of DCQD on downstream effectors in the JAK2/STAT3 pathway. (a) Real-time PCR was performed to detect the mRNA expression of Bax and Bcl-XL. (b) Western blot was performed to detect the protein level of p-STAT3, Bax, and Bcl-XL. GAPDH was as the loading control for band density normalization. Data are presented as the means ± SD, *n* = 3. All bar graphics: #*P* < 0.05, ##*P* < 0.01 vs. the model group.

According to the protein expression of p-STAT3 in different groups (Figure 5(b)), DCQD did suppress the activation of JAK2/STAT3 signaling pathway, which was consistent with our previous experimental results (Figure 4(b)). Since Bax and Bcl-XL are two of the crucial downstream effectors of STAT3, the levels of Bax and Bcl-XL were tested to further verify the inhibitory effect of DQCD on JAK2/STAT3 pathway. As shown in Figure 5(a), the mRNA expression of Bax was significantly increased in CER-treated groups (*P* < 0.01). Similarly, a noticeable increase was also found in protein expression of Bax in CER-treated groups (Figure 5(b)) (*P* < 0.01). DCQD counteracted the effect of CER, and the expression of Bax was significantly decreased compared to model group (*P* < 0.05). By contrast, the protein and mRNA expression of Bcl-XL showed an opposite trend against Bax. These results further confirmed that DCQD did suppress the JAK2/STAT3 pathway and regulated the expression of JAK2/STAT3 downstream effectors, such as Bax and Bcl-XL. Furthermore, given that no significant difference was found between DG and control groups (*P* > 0.05), the side effect of DCQD could be excluded.

The amelioration of DCQD was mediated by suppressing the activation of JAK2/STAT3 signaling pathway.

**Figure 6:**
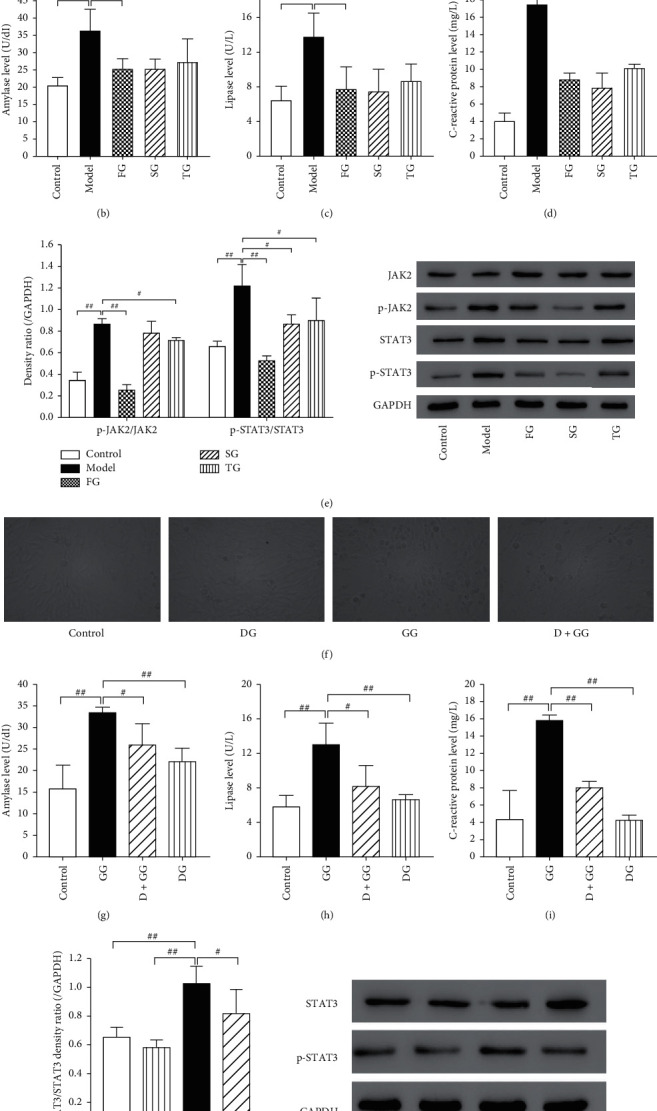
The amelioration of DCQD was mediated by suppressing the activation of JAK2/STAT3 signaling pathway. (a and f) The morphological changes of cells were assessed by using an optical microscope (400×). The releases of amylase, lipase, and C-reactive protein were detected by amylase assay kit (b and g), lipase assay kit (c and h), and C-reactive protein assay kit (d and i), respectively. (e) Western blot was performed to detect the protein level of JAK2, p-JAK2, STAT3, and p-STAT3. GAPDH was as the loading control for band density normalization. (j) Western blot was performed to detect the protein level of STAT3 and p-STAT3. GAPDH was as the loading control for band density normalization. Data are presented as the means ± SD, *n* = 3. Micrographs from one representative experiment out of three independent experiments are shown. All bar graphics: #*P* < 0.05, ##*P* < 0.01 vs. the model group.

To test whether the amelioration effect of DCQD depended on the suppression of JAK2/STAT3 signaling pathway, selective JAK2 and STAT3 inhibitors as well as STAT3 activator were used. The inhibitors (Fedratinib and Stattic) tend to suppress the expression of JAK2/STAT3 signaling pathway, while the activator (Garcinone D) tends to overexpress it. Compared to control group, no significant morphological changes of cells were observed under optical microscopy in FG, SG, and TG (Figure 6(a)). In addition, Figures 6(b)–6(d) show that the results of control, model, and TG were in line with our previous experimental results (Figure 2). Fedratinib (JAK2 inhibitor) and Stattic (STAT3 inhibitor) significantly reduced the releases of amylase, lipase, and C-reactive protein of cells (*P* < 0.01). Notably, the effects of Fedratinib and Stattic on improving cells injury and reducing the reflective markers of severity of AP were more potent than that of DCQD. Further Western blotting analysis demonstrated our findings (Figure 6(e)). Stimulation with CER significantly increased the p-JAK2/JAK2 and p-STAT3/STAT3 levels compared to that in control group (*P* < 0.01). This increase was dramatically counteracted by DCQD and Fedratinib (*P* < 0.05 and *P* < 0.01, respectively) while Stattic only decreased the p-STAT3/STAT3 level (*P* < 0.05), as we expected. We reasoned that JAK2/STAT3 signaling pathway played an important role in cells injury induced by CER. Furthermore, we also used Garcinone D (STAT3 activator) to demonstrate the role of JAK2/STAT3 in exhibited effects of DCQD. As shown in Figures 6(g)–6(i), like CER, GG had a similar damage effect on cells. The releases of amylase, lipase, and C-reactive protein of cells in GG were significantly increased (*P* < 0.01). And the cells exhibited morphological features of injury when treated with Garcinone D compared to control group. However, pretreatment of DCQD (C/4) markedly reversed the increase of amylase, lipase, and C-reactive protein in cells. Besides, the morphological features showed some improvement (Figure 6(f)). The volume of cells tended to be normal, and the secretions such as trypsin decreased significantly. Western blotting assays confirmed the above findings as well (Figure 6(j)). In GG group, the p-JAK2/JAK2 and p-STAT3/STAT3 levels were markedly increased compared with that in the control group (*P* < 0.01). Yet, this increase was significantly reversed by DCQD (*P* < 0.05). Moreover, no discernible difference was observed between the groups treated with DCQD alone and control group (*P* > 0.05), which ruled out the possible effect of DCQD. Overall, we reasoned that the effect of DCQD is, at least, partially medicated through the JAK2/STAT3 signaling pathway.

## 4. Discussion

DCQD reportedly inhibits the cytokine's activity, promotes the gastrointestinal motility, and regulates the inflammatory response in patients with AP [23]. However, the molecular mechanism underlying DCQD effect on pancreatic acinar cells is not fully understood. Here, we found that DCQD protected AR42J pancreatic acinar cells by inhibiting CER-induced inflammation and reducing the secretion of pancreatic biochemical markers, such as amylase, lipase, and C-reactive protein. The mechanisms mediating these effects may be related to the suppression of the JAK2/STAT3 signaling pathway. Additionally, DCQD could significantly regulate the mRNA and protein expression of JAK2/STAT3 downstream effectors, Bax and Bcl-XL.

At present, AR42J is one of the cell lines with the characteristics of normal pancreatic acinar cells, which has been used to study the growth, secretion, and proliferation of pancreatic exocrine cells [24]. CER is an ortholog of the intestinal hormone cholecystokinin, which is one of the most characterized and widely used agents in experimental animal models of pancreatitis. CER has been reported to simulate the characteristics of human pancreatitis by inducing the death of acinar cells, the formation of edema, and the infiltration of inflammatory cells into the pancreas in vivo and in vitro [25, 26]. In this research, the rat pancreatic acinar cell line AR42J was used as an in vitro model to evaluate the effect of DCQD against pancreatic acinar cell injury induced by CER. In agreement with previous studies, our results showed that 10^−7^M CER notably induced cell injury [15].

Generally, AP is recognized as an excessive inflammatory response characterized by the dysregulation of digestive enzyme production and secretion [25]. Indeed, the death of acinar cells due to uncontrolled inflammation may be one of the crucial factors resulting in AP. Once pancreatic acinar cells are injured or dead, cytokines will be released, which can trigger an inflammatory response and ultimately lead to devastating consequences [27]. The release of these inflammatory factors from damaged acinar cells, as one of the early events in the development of AP, is associated with the degree of pancreatic inflammation [28]. IL-6, a multifunctional cytokine most closely associated with STAT transcription factor activity [29], is the most credible candidate in assessing the severity of AP and predicting the risk of early complications, with a high sensitivity range (about 89%–100%) [30]. TNF-*α* can regulate not only leukocyte adhesion molecules but also other proinflammatory cytokines. It can act as a priming activator of immune cells as well. Recently, a number of studies have shown that TNF-*α* plays a pivotal role in the pathogenesis of AP, most of which come from both animal models and in vitro studies. In particular, it promotes the systemic progression of inflammation and terminal organ dysfunction, which is usually observed in severe diseases [31]. Another major cytokine, IL-1*β*, can drive the excessive systemic inflammatory response, too. Since the levels of IL-1*β* and IL-6 of the pancreatic acinar cells stimulated with CER or the activated neutrophils were similar at the late stage of stimulation, it has similar accuracy to IL-6 in predicting AP upon admission and also has been used as a biomarker of disease severity [32]. Moreover, in CER-stimulated pancreatic acinar cells, IL-1*β* could be detected earlier and more expressed at early stage of stimulation [33, 34]. In contrast, IL-10, an anti-inflammatory cytokine, acts as potent suppressant. Once activated, it can prevent the extracellular killing function of macrophages [35]. IL-10 is likely a primary factor in the negative feedback system that hinders the production of proinflammatory cytokines like IL-6 and TNF-*α* in various cells [36, 37]. Interestingly, in previous studies, the level of IL-10 showed different trends in various AP models. In vivo experiment, Yuan et al. found no change in the concentration of IL-10 in kidney tissues of rats with sever AP [38]. But in the mice with pancreatitis-induced lung injury, Piao et al. found that the level of IL-10 in serum was significantly decreased [39]. In vitro studies, an increase in IL-10 production was observed in the supernatant of pancreatic acinar cells after stimulation with TNF-*α* [19]. A previous study also found, in CER-stimulated AR42J pancreatic acinar cells, that the expression of IL-10 was markedly increased in gene level [40]. However, another research demonstrated that, in the supernatant of pancreatic acinar cells treated with lipopo1ysaccharide, the levels of IL-10 in culture supernatant decreased with the lipopo1ysaccharide concentration. We suspected that a reason for these discrepancies is mainly due to the different kinds of AP model or differences in detection methods and levels. Previous study has provided evidence for the effect of DCQD on the control of acute kidney injury in rats with severe acute pancreatitis by cytokine inhibition. This suggested that long-term treatment with DCQD can reduce exacerbations and complication in AP [38].This study also noticed very high levels of proinflammatory factors, including TNF-*α*, IL-1*β*, and IL-6 as well as a lower level of anti-inflammatory factor IL-10 in the supernatant of CER-induced cells. These changes in cytokine levels were noticeably mitigated by DCQD pretreatment, which means that DCQD may play a critical role in the regulation of the inflammatory response in AP. These results combined with the increased cell viability and improvement of cell morphology and structure indicated that DCQD might exert a therapeutic effect for AP, at least, in part via creating a balance between proinflammatory and anti-inflammatory factors.

In the diagnosis of AP, serum amylase and lipase remain important tests with a high specificity. However, they cannot reflect its severity [41]. In this respect, C-reactive protein has been established as a prognostic variable in human AP [42]. Past researches have proved both the diagnostic value of amylase and lipase as well as the prognostic value of C-reactive protein [20, 21]. In this study, DCQD effectively reversed the CER-induced increase in amylase, lipase, and C-reactive protein levels. Moreover, DCQD showed a dose-effect relationship.

The JAK2/STAT3 signaling pathway is a pleiotropic cascade responsible for the transcription of various genes involved in immunity and inflammation. An increasing body of evidence has shown the important role of JAK2/STAT3 pathway in the development of AP [43, 44]. JAK2 can be activated by numerous cytokines or growth factors. Ligand binding induces phosphorylation of tyrosine kinases and isomerization of JAK-related receptors subunits. Activated JAK in turn phosphorylates receptors for recruitment of STAT protein [45]. STAT3 is one of the pivotal JAK2 effectors and its transcriptional activity is regulated by phosphorylation of JAK2. The phosphorylated STAT3 is released from the receptor complex to form a dimer. Then, these dimers will translocate to the nucleus, where they directly bind to the promoter regions of specific target genes, thereby regulating the transcription of the target genes, such as Bcl-2 family [46]. Proapoptotic protein Bax and antiapoptotic protein Bcl-XL are two of the representative members in Bcl-2 family [47]. Bax and Bcl-XL can regulate each other's functions through heterodimers. And their relative concentrations may play an important role in regulating apoptosis [47].Of note, study has also shown that DCQD, as an inhibitor of the JAK2/STAT3 pathway, exerts marked effects in AP models in vivo [14]. This study investigated the role of JAK2/STAT3 pathway in CER-induced AR42J cells and whether DCQD can improve the cell injury through this pathway. Firstly, consistent with previous study [25], the results showed that CER induced the phosphorylation of JAK2 and STAT3 in AR42J cells. Pretreatment with JAK2 inhibitor, Fedratinib, or STAT3 inhibitor, Stattic, reversed the activated effect of CER on the phosphorylation of JAK2 and STAT3. Furthermore, the overexpression of JAK2/STAT3 pathway, via STAT3 activator Garcinone D, did exert damage on cells, which bore a resemblance to CER. Based on these, we reasoned that the JAK2/STAT3 signaling pathway is a potential therapeutic target in the treatment of AP. Next, we also found that DCQD exhibited dose-dependent inhibitory effects on the JAK2/STAT3 signaling pathway. DCQD could not only inhibit the nuclear translocation of p-STAT3, but also decrease the mRNA expression of JAK2 and STAT3 as well as the ratio of p-JAK2/JAK2 and p-STAT3/STAT3 in protein level. Moreover, DCQD could also regulate the expression of JAK2/STAT3 downstream effectors, such as Bax and Bcl-XL. In a word, the present study provided in vitro evidence that the JAK2/STAT3 pathway is activated in AP and that the protective effect of DCQD in AP is due to inhibition of the overexpression of JAK2/STAT3.

However, some questions have yet to be solved: due to the fact that the molecular targets of DCQD may not be only dependent on the JAK2/STAT3 signaling pathway, the possibility of other mechanisms cannot be excluded. Moreover, since DCQD succeeded in regulating the apoptotic Bcl-2 family members, Bax and Bcl-XL, it is not clear whether it could further modulate pancreatic acinar cell apoptosis. Further studies are required to answer these questions and explore the mechanism of action of DCQD in detail.

## 5. Conclusion

The activation of JAK2/STAT3 pathway may play an important role in the pathogenesis of cerulein-stimulated AR42J pancreatic acinar cell injury. DCQD could improve inflammatory cytokines and cell injury, which might be mediated by suppressing the activation of JAK2/STAT3 signaling pathway.

## Figures and Tables

**Table 1:** Primer sequences of mRNA.

Gene	Product size, bp	Primer (5′–3′)
JAK2	206	Primer F: CAGGGAATCCACCTTTCATC
		Primer R: CAGAGCACTCAGAGGCTTGTC
STAT3	242	Primer F: GCCATCCTAAGCACAAAGC
		Primer R: TCAGGGTAGAGGTAGACCAGTG
Bcl-XL	133	Primer F: ATGGGGTAAACTGGGGTCG
		Primer R: TGGTCATTCAGGTAGGTGGC
	298	Primer F: GCGATGAACTGGACAACAAC
		Primer R: CCGAAGTAGGAAAGGAGGC
Bax	237	Primer F: GGAGTCTACTGGCGTCTTCAC
		Primer R: ATGAGCCCTTCCACGATGC

## Data Availability

The datasets used and analyzed during the current study are available from the corresponding author on reasonable request.

## Abbreviations

DCQD: Da Cheng Qi Decoction
AP: Acute pancreatitis
SIRS: Systemic inflammatory response syndrome
MODS: Multiple organ dysfunction syndromes
TNF-*α*: Tumor necrosis factor-*α*
IL-6: Interleukin-6
IL-10: Interleukin-10
IL-1*β*: Interleukin-1*β*
JAK2: Janus kinase2
STAT3: Signal transducers and transcription 3
T.C.M.: Traditional Chinese Medicine
CER: Cerulein
FBS: Fetal bovine serum
PBS: Phosphate buffered saline
CCK-8: Cell counting kit-8
ELISA: Enzyme-Linked immunosorbent assay
BSA: Bovine serum albumin
p-STAT3: Phosphorylated-signal transduction and activators of transcription 3
p-JAK2: Phosphorylated-Janus kinase 2
Real-time PCR: Real-time polymerase chain reaction
cDNA: Complementary DNA
GAPDH: Glyceraldehyde-3-phosphate dehydrogenase
Bcl-XL: B-cell lymphoma-extra large
Bax: Bcl2-associated X
BCA: Bicinchoninic acid
SDS-PAGE: Sodium dodecylsulfate–polyacrylamide gel electrophoresis
PVDF: Polyvinylidene fluoride
HRP: Horseradish peroxidase
TBST: Tris-buffered saline Tween
RIPA: Radio-immunoprecipitation Assay
SD: Standard deviation
ANOVA: A one-way analysis of variance
ECL: Enhanced chemiluminescence.

## Supplementary Materials

Supplementary Materials Figure 1. Graphical abstract. Da Cheng Qi Decoction alleviates cerulein-stimulated AR42J pancreatic acinar cell injury via the JAK2/STAT3 signaling pathway. Additional file: Figure 2. The HPLC-UV fingerprints of DCQD. The HPLC-UV fingerprint shows that the active ingredients of DCQD.9 major compounds were detected in DCQD by the HPLC-UV method. S2: DCQD, ST: mixed standard solution, 1: naringin, 2: hesperidin, 3: aloe-emodin, 4: rhein, 5: honokiol, 6: magnolol, 7: emodin, 8: chrysophanol, and 9: physcion.
