# Supplementary material for: Da Cheng Qi Decoction Alleviates Cerulein-Stimulated AR42J Pancreatic Acinar Cell Injury via the JAK2/STAT3 Signaling Pathway
Source: Evid Based Complement Alternat Med. 2021 Apr 9;2021:6657036. doi: 10.1155/2021/6657036 (PMC8053057; doi:10.1155/2021/6657036)
Supplement: Supplementary Materials — Figure 1. Graphical abstract. Da Cheng Qi Decoction alleviates cerulein-stimulated AR42J pancreatic acinar cell injury via the JAK2/STAT3 signaling pathway. Additional file: Figure 2. The HPLC-UV fingerprints of DCQD. The HPLC-UV fingerprint shows that the active ingredients of DCQD.9 major compounds were detected in DCQD by the HPLC-UV method. S2: DCQD, ST: mixed standard solution, 1: naringin, 2: hesperidin, 3: aloe-emodin, 4: rhein, 5: honokiol, 6: magnolol, 7: emodin, 8: chrysophanol, and 9: physcion. [file 6657036.f1.zip › 6657036.f1/The gray value and statistical analysis of WB detection.pdf]

Fig.4 B	Control	Model	LG	MG	HG
gray value：P/T(JAK2)	0.604125939	1.0559214	0.881322236	0.974624043	0.804203377
	0.886388125	1.101538506	0.98662156	0.908739529	0.810155353
	0.693063592	1.163840788	0.9964313	0.858927011	0.905196165
mean	0.727859219	1.107100231	0.954791699	0.914096861	0.839851632
SD	0.14431229	0.054174239	0.063815196	0.05803427	0.056668223
P Value(vs Model)	0.000228		0.048301	0.017271	0.002751

	Control	Model	LG	MG	HG
gray value：P/T(STAT3)	0.4084965	1.151587706	0.957795063	0.787303035	0.601902525
	0.528497742	0.907597366	0.759913387	0.765758763	0.700339957
	0.394198303	0.904050806	0.851415296	0.620962101	0.504638243
mean	0.443730848	0.987745293	0.856374582	0.724674633	0.602293575
SD	0.073757571	0.141902772	0.099034011	0.09046135	0.097851443
P Value(vs Model)	0.000072		0.149666	0.010778	0.001012

Fig.5 B	Control	Model	TG	DG
gray	0.269811	0.773631	0.815052	0.281939
value:p-	0.299587	0.892313	0.478521	0.253777
STAT3/GAPD	0.20517	1.14767	0.635255	0.229998
mean	0.258189333	0.937871333	0.642942667	0.255238
SD	0.04826945	0.191135985	0.16839716	0.026001303
P Value(vs	0.000212		0.024204	0.000205

	Control	Model	TG	DG
gray	0.127373	1.14478	0.858829	0.266318
value:Bax/	0.0418142	0.953728	0.676896	0.522856
GAPDH	0.072223	1.3727	0.975371	0.238516
mean	0.080470067	1.157069333	0.837032	0.342563333
SD	0.043371507	0.20975618	0.150426604	0.156755612
P Value(vs	0.000025		0.033153	0.00018

	Control	Model	TG	DG
gray	1.33391	0.296371	0.660103	0.699378
value:Bcl-	1.08512	0.390566	0.550856	0.793938
XL/GAPDH	1.13183	0.288544	0.622429	0.584986
mean	1.18362	0.325160333	0.611129333	0.692767333
SD	0.132233767	0.056778001	0.05549314	0.10463274
P Value(vs	0.000003		0.005557	0.001302

Fig.6 E	Control	Model	FG	SG	TG
gray value: P/T(JAK2)	0.309419	0.822907	0.272299	0.7387	0.700836
	0.428176	0.886535	0.22813	0.710692	0.706905
	0.33989	0.909484	0.295386	0.903995	0.746833
mean	0.359161667	0.872975333	0.265271667	0.784462333	0.718191333
SD	0.061679446	0.044853012	0.034174259	0.104461265	0.024989338
P Value(vs Model)	0.000001		0.000001	0.105224	0.010971

	Control	Model	FG	SG	TG
gray value: P/T(STAT3)	0.692519	1.21045	0.550517	0.957979	1.09329
	0.684773	1.42552	0.55667	0.806109	0.936441
	0.618946	1.02578	0.483983	0.857852	0.694072
mean	0.665412667	1.220583333	0.53039	0.87398	0.907934333
SD	0.040427261	0.200062566	0.040307221	0.077208863	0.201129872
P Value(vs Model)	0.00048		0.000088	0.01	0.01699

Fig.6 J	Control	DG	GG	D+GG
gray value: P/T(STAT3)	0.721834	0.635665	1.11973	0.983136
	0.670028	0.54369	1.07864	0.65615
	0.592875	0.586675	0.907605	0.825467
mean	0.661579	0.588676667	1.035325	0.821584333
SD	0.064893336	0.04602016	0.112500648	0.163527574
P Value(vs GG	0.002683	0.000911		0.040038
