# Supplementary figures and images for: Da Cheng Qi Decoction Alleviates Cerulein-Stimulated AR42J Pancreatic Acinar Cell Injury via the JAK2/STAT3 Signaling Pathway
Source: Evid Based Complement Alternat Med. 2021 Apr 9;2021:6657036. doi: 10.1155/2021/6657036 (PMC8053057; doi:10.1155/2021/6657036)

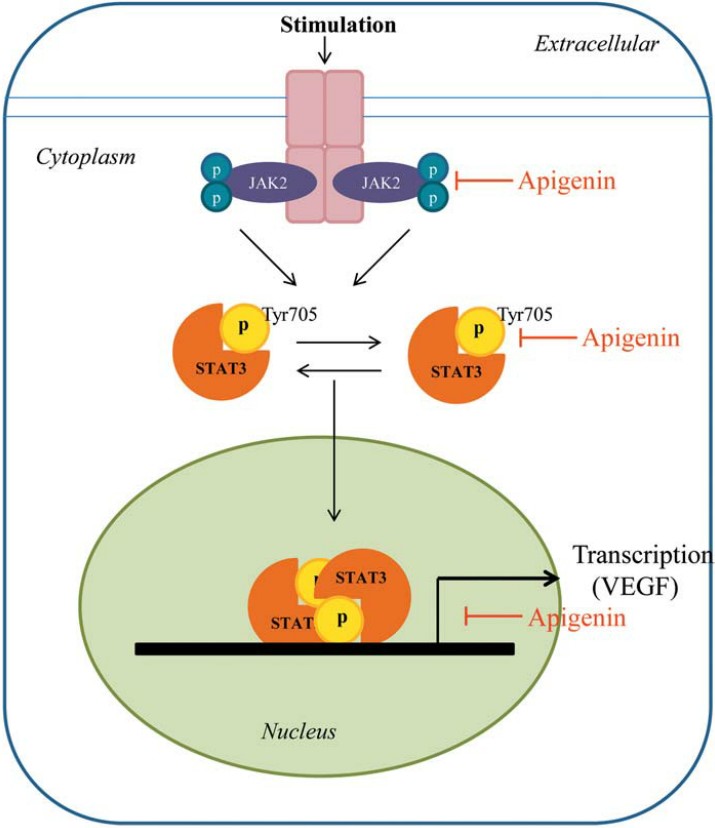

Supplement: Supplementary Materials — Figure 1. Graphical abstract. Da Cheng Qi Decoction alleviates cerulein-stimulated AR42J pancreatic acinar cell injury via the JAK2/STAT3 signaling pathway. Additional file: Figure 2. The HPLC-UV fingerprints of DCQD. The HPLC-UV fingerprint shows that the active ingredients of DCQD.9 major compounds were detected in DCQD by the HPLC-UV method. S2: DCQD, ST: mixed standard solution, 1: naringin, 2: hesperidin, 3: aloe-emodin, 4: rhein, 5: honokiol, 6: magnolol, 7: emodin, 8: chrysophanol, and 9: physcion. [file 6657036.f1.zip › 6657036.f1/Graphic Abstract.jpg]
